# Clinical Validation of the Proenkephalin (*PENK*) Methylation Urine Test for Monitoring Recurrence of Non–muscle-invasive Bladder Cancer

**DOI:** 10.1016/j.euros.2024.02.010

**Published:** 2024-03-07

**Authors:** Hyunho Han, Tae Jeong Oh, Ji Eun Heo, Jongsoo Lee, Won Sik Jang, Seung Hwan Lee, Won Sik Ham, Jaehee Hwang, Sungwhan An, Young-Deuk Choi

**Affiliations:** a Department of Urology, Urological Science Institute, Yonsei University College of Medicine, Seoul, Republic of Korea; b Genomictree, Inc., Daejeon, Republic of Korea

**Keywords:** *PENK* methylation, Bladder cancer, Surveillance, Urine-derived DNA, Molecular biomarker

## Abstract

**Background and objective:**

To assess the effectiveness of a urine-based proenkephalin (*PENK)* methylation test using linear target enrichment-quantitative methylation-specific polymerase chain reaction (me*PENK* test) for detection of non–muscle-invasive bladder cancer (NMIBC) recurrence compared to cytology and the NMP22 test.

**Methods:**

We first conducted a retrospective case-control study involving 54 patients with primary BC and 29 healthy individuals. We then prospectively enrolled 186 patients (January to December 2022) undergoing cystoscopy surveillance after transurethral resection of bladder tumor, of whom 59 had recurrent tumors. We analyzed voided urine samples for *PENK* methylation levels in urinary DNA. Cystoscopy with histology was used as the reference standard for assessing the diagnostic accuracy of the me*PENK* test in detecting BC recurrence. We calculated the sensitivity and specificity using receiver operating characteristic curve analysis. Survival differences were determined using the Kaplan-Meier method and Cox proportional-hazards model. A *p* < 0.05 was considered statistically significant.

**Key findings and limitations:**

In the case-control study, the *PENK* test had sensitivity of 83.3% and specificity of 100%. For NMIBC patients undergoing cystoscopy surveillance, the sensitivity was 76.3% (95% confidence interval [CI] 63.4–86.4%) and the specificity was 85% (95% CI 77.6–90.7%), outperforming cytology (sensitivity: 28.8%, 95% CI 17.8–42.1%; *p* < 0.001; specificity: 97.6%, 95% CI 93.2–99.5%) and the NMP22 test (sensitivity: 54.2%, 95% CI 40.7–67.2%; *p* = 0.016; specificity 81.9%, 95% CI 74.1–88.2%). In the high-risk group, the me*PENK* test had sensitivity of 89.7% (95% CI 75.8–97.1%) and a negative predictive value of 96.9%. For the group with low/intermediate risk, the sensitivity was 41.7%. In the group with negative cystoscopy, recurrence-free survival was shorter for patients with positive than for those with negative me*PENK* results (245 vs 503 d), with a hazard ratio of 9.4 (*p* < 0.001). The main study limitation is the small sample size.

**Conclusions and clinical implications:**

The me*PENK* test showed good performance for detection of NMIBC recurrence and has potential for use for prognosis and prediction.

**Patient summary:**

We found that a test used to analyze urine samples showed good performance in detecting recurrence of NMIBC. This noninvasive me*PENK* test may help in personalized follow-up care for patients with NMIBC.

## Take Home Message

The me*PENK* test showed good performance in detecting recurrence of non–muscle-invasive bladder cancer and outperformed cytology and the NMP22 test in diagnostic accuracy. The me*PENK* test offers promising clinical utility for NMIBC management and monitoring, particularly for patients with high-risk disease.

## 1 Introduction

Bladder cancer (BC) is the ninth most common cancer worldwide, with approximately 549 000 new cases reported annually [1]. Some 75% of these cases are non–muscle-invasive BC (NMIBC), which has a high recurrence rate after transurethral resection of bladder tumor (TURBT) and potential for progression to muscle-invasive disease [2]. Cystoscopy in conjunction with urine cytology is the standard for NMIBC surveillance. Although urine cytology has high specificity, its sensitivity is limited, especially for low-grade tumors [3], which can lead to overdiagnosis and unnecessary invasive procedures. Accurate and precise surveillance methods are essential for prompt detection and treatment of recurrent NMIBC [4].

Urine-based biomarker tests for BC surveillance, such as the BTA, NMP22, and UroVysion tests, have been approved by the US Food and Drug Administration. Tests such as CxBladder test and Xpert Bladder Cancer Monitor, which detect multiple mRNA biomarkers in urine samples, have been also developed for BC recurrence surveillance [5], [6], [7], [8].

Aberrant DNA methylation, a major epigenetic modification, often results in inactivation of tumor suppressor and other cancer-related genes [9], [10]. Specific aberrant methylation sites on relevant genes are potential biomarkers for early detection of BC [11], [12]. The EpiCheck test uses a panel of 15 DNA methylation sites as biomarkers in urine and is a promising BC detection approach [13].

We previously identified proenkephalin (*PENK)* methylation as a specific diagnostic biomarker for BC and demonstrated its clinical validity for BC detection by measuring *PENK* methylation in urine via quantitative methylation-specific polymerase chain reaction (PCR) qMSP) following linear target enrichment (LTE) (me*PENK*-LTE/qMSP test) [14], [15]. In the present study we further optimized this urine-based me*PENK* test using a closed single-tube system. The method involves two consecutive rounds of PCR: (1) a linear amplification step to enrich methylated *PENK* target DNA from a bisulfite-treated samples; and (2) qMSP for amplification of the me*PENK* target region and the control gene. Our aim was to evaluate the clinical effectiveness of the me*PENK* test for BC recurrence surveillance after TURBT and to compare its performance with the NMP22 test and cytology.

## 2 Patients and methods

### 2.1 Study design and patients

We performed a retrospective case-control analysis to determine the optimal cutoff value for the me*PENK* test for detection of BC. The study population comprised 54 patients with primary BC and 29 healthy individuals.

**Fig. 1:**
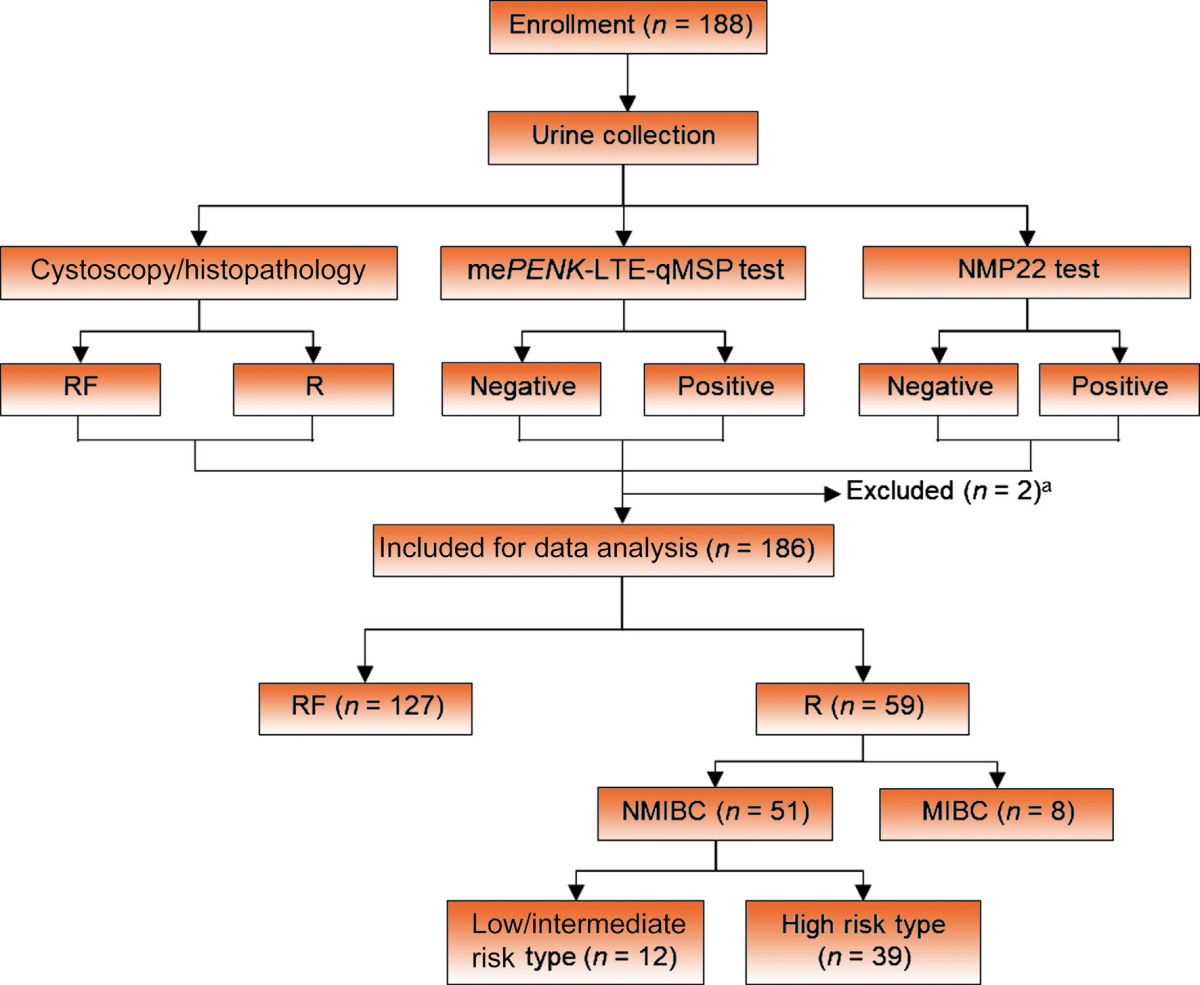
Study workflow. MIBC = muscle-invasive bladder cancer; NMIBC = non–muscle-invasive bladder cancer; RF = recurrence-free; R = recurrence. ^a^ Excluded because of insufficient information.

In an additional prospective pilot study, 188 patients with a history of BC and scheduled for cystoscopy surveillance after TURBT were enrolled between January and December 2022 (Fig. 1). This study received institutional review board approval (#4-2021-0406) and all patients provided written consent.

### 2.2 Sample collection, DNA isolation, and bisulfite treatment

A sample of voided urine (20 ml) was collected before cystoscopy using a Urine Collection Kit I (Genomictree Inc., Seoul, Republic of Korea). Genomic DNA was extracted from urine sediment and was chemically modified with sodium bisulfite for methylation analysis.

### 2.3 *PENK* methylation assessment using the me*PENK* test

The me*PENK* test was developed to assess *PENK* methylation in urine-derived DNA. This assay, conducted in a closed tube system, involves a two-round PCR process for accurate and continuous amplification of *PENK* methylated DNA and control DNA targets (Supplementary Fig. 1). In the first PCR round, a high annealing temperature is applied to facilitate unidirectional DNA synthesis. A specific primer, featuring a unique tag sequence (UTS) at its 5′ end, was designed to anneal to the *PENK* methylation site under the elevated temperature (70°C) conditions, preventing other primers with regular melting temperatures from initiating DNA synthesis. The template DNA synthesis occurs in one direction only, replicating in each PCR cycle.

In the second PCR round, the reaction is conducted at a normal temperature (60°C). Two distinct sets of primers and probes are used for amplification of the *PENK* methylation target and of the control target. The forward primer for the *PENK* methylation target was designed to bind specifically to the *PENK* methylation site, and the reverse primer contained a UTS. For the control target, a primer set specific to a DNA region of *COL2A1* was used, with annealing at the normal temperature. Probes were designed to bind to the internal sites of each target PCR product; generation of discernible signals indicated PCR product formation.

Genomic DNA isolated from the sediment from 10 ml of urine was used as the input for bisulfite treatment. Each 25-μl reaction mixture contained 18 μl of bisulfite-converted DNA, 200 nmol/l *PENK* methylation–specific forward primer, 40 nmol/l *PENK* methylation-specific reverse primer with a UTS at the 5′ end, 160 nmol/l *PENK* probe, 200 nmol/l *COL2A1* forward primer, 200 nmol/l *COL2A1* probe, 200 nmol/l *COL2A1* reverse primer, 200 nmol/l reverse primer for the UTS, and 5 μl of 5× Fast qPCR PreMIX TaqMan Probe (Enzynomics Inc., Daejon, Republic of Korea). *COL2A1,* which lacks the CpG dinucleotide, was used as a control. The primer and probe sequences are provided in Supplementary Table 1.

The entire me*PENK* test procedure was performed in a single closed tube on an AB7500 FAST Real-Time PCR system (Thermo Fisher Scientific, Waltham, MA, USA). The specific thermal cycling conditions were 95°C for 5 min, followed by 15 cycles at 95°C for 15 s and 70°C for 45 s, then 35 cycles at 95°C for 15 s and 60°C for 45 s. The relative level of *PENK* methylation in each sample was calculated as 35−ΔC_T_ , where ΔC_T_ is the difference in cycle threshold between the amplified *PENK* target and *COL2A1* [12]. Higher 35−ΔC_T_ values indicated higher levels of *PENK* methylation. If the *PENK* C_T_ was undetectable, the value was set to 20, which was the value closest to the lowest 35−ΔC_T_ result for all tests.

### 2.4 NMP22 test

The Alere NMP22 test (Abbott, Abbott Park, IL, USA), an enzyme immunoassay method, was used for NMP22 detection in urine samples. The detection threshold was 10 IU/ml.

### 2.5 Reference standard

Cystoscopy with a pathology result (TURBT under general anesthesia) was the reference standard. Results were classified as recurrence or recurrence-free. Recurrence events were subcategorized as progression to muscle-invasive disease or progression-free.

### 2.6 Survival analysis

Recurrence-free survival (RFS) was defined as the time from diagnosis to the occurrence of recurrence. Patients who were recurrence-free at the time of diagnosis had follow-up cystoscopy at median follow-up of 223 d. Survival curves were estimated using the Kaplan-Meier method, and differences in survival distributions were tested for significance using the log-rank test. The hazard ratio (HR) and its 95% confidence interval (CI) were estimated using a Cox proportional-hazards model.

### 2.7 Data analysis

Diagnostic accuracy was evaluated using receiver operating characteristic (ROC) analysis and calculation of the area under the ROC curve (AUC). Statistical significance was set at a *p* value of <0.05. Sensitivity and specificity were calculated in a binary manner for comparison of the clinical performance of the me*PENK* test to NMP22 and cytology tests using the McNemar test. CIs (95%), negative predictive values (NPVs), and positive predictive values (PPVs) were computed using MedCalc version9.3.2.0 (MedCalc Software Ltd., Ostend, Belgium). The incidence of recurrent BC was estimated on the basis of this study.

## 3 Results

### 3.1 Study design and patient demographics

We included 54 patients with primary BC and 29 healthy individuals in a case-control study to determine optimal cutoff values for the me*PENK* test. A total of 188 participants were recruited for a prospective study, of whom two were excluded because of insufficient data. Of the 186 participants with results that could be fully evaluated, 59 (31.7%) had a recurrent bladder tumor at evaluation. Among these recurrent tumors, nine (15.3%) were stage Ta, 12 (20.3%) were carcinoma *in situ* (CIS), 30 (50.8%) were stage T1, and eight (13.6%) were stage T2–4. Demographic information for participants who were fully evaluated is presented in .Table 1

**Table 1:** Demographic and clinical characteristics of the study population

Parameter	Case-control study		ProspectiveBC cohort
	Healthy controls	BC patients	
Participants (*n*)	29	54	186
Sex, *n* (%)			
Male	23 (79.3)	45 (83.3)	157 (84.4)
Female	6 (20.7)	9 (16.7)	29 (15.6)
Mean age, yr (range)	35.2 (26–57)	72.9 (42–90)	69.1 (45–93)
Recurrence			
Yes			59 (31.7)
No			127 (68.3)
pT stage at recurrence, *n* (%)			
Ta		22 (40.7)	9 (15.3)
Carcinoma *in situ*		1 (1.85)	12 (20.3)
T1		27 (50.0)	30 (50.8)
T2–4		4 (7.4)	8 (13.6)
Grade			
Low		11 (20.4)	12 (20.3)
High		42 (77.8)	41 (69.5)
Unknown		1 (1.85)	6 (10.2)
Progression a			
Yes			17 (28.8)
No			42 (71.2)

BC = bladder cancer.

a Recurrence with an increase in tumor stage or grade in comparison to the previous visit.

### 3.2 Determination of the me*PENK* cutoff

**Fig. 2:**
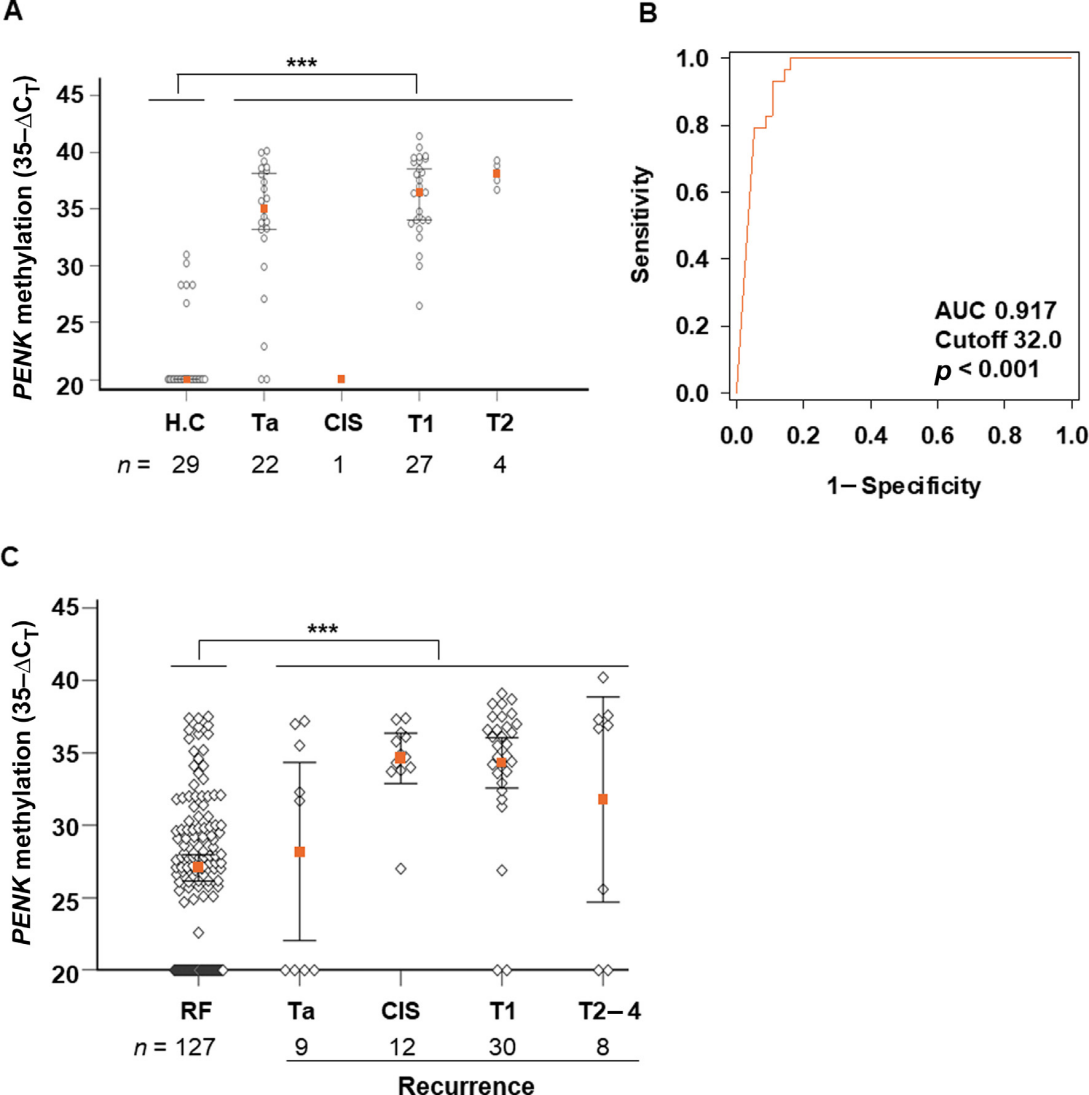
Cutoff determination for the me*PENK* test and clinical validation of the test for detection of recurrent BC in the prospective study. (A) Distribution of *PENK* methylation values (35−ΔC_T_) for 83 urine samples. Error bars denote the 95% confidence intervals. (B) Receiver operating characteristic curve for detection of all bladder cancer cases. (C) Scatter plot of *PENK* methylation values (35−ΔC_T_) for the 186 urine samples evaluated. Error bars denote the 95% confidence intervals. AUC = area under the curve; CIS = carcinoma *in situ*; C_T_ = cycle threshold; H.C = healthy controls. * *p* < 0.001 (Kruskal-Wallis test).

We determined a cutoff value for the test via retrospective case-control analysis for 54 patients with primary BC and 29 healthy individuals. Our findings demonstrated significantly higher *PENK* methylation levels in samples from BC patients in comparison to the healthy control subjects (*p* < 0.001; Kruskal-Wallis test; Fig. 2A). Remarkably, among the 29 healthy subjects, only six exhibited real-time PCR signals within the specified range (35−ΔC_T_: 26.7–31.0).

The ROC analysis for discrimination between all BC cases and healthy individuals identified a 35−ΔC_T_ cutoff value of 32.0. This threshold exhibited 100% specificity (29/29; 95% CI 88.1–100%) and sensitivity of 83.3% (45/54; 95% CI 70.7–92.1%), with an AUC of 0.917 (95% CI 0.835–0.966; Fig. 2B). Sensitivity values for BC stages were 77.3% (17/22) for Ta, 88.9% (24/27) for T1, 0% (0/1) for CIS, and 100% (4/4) for T2.

### 3.3 Clinical performance of the me*PENK* test for detection of recurrent BC

The clinical performance of the me*PENK* test was evaluated prospectively. Before surveillance cystoscopy, voided urine samples were collected from 186 patients with a history of BC. Using the cutoff value of 32.0 determined in the case-control study, the test had overall sensitivity of 76.3% (45/59; 95% CI 63.4–86.4%) and specificity of 85.0% (108/127; 95% CI 77.6–90.7%) for detection of all recurrent BCs. The AUC was 0.807 (95% CI 0.742–0.861; Fig. 2C). The NPV was 88.5% (95% CI 82.9–92.5%) and the PPV was 70.3% (95% CI 60.4–78.6). Results for sensitivity by stage and grade are presented in Table 2.

**Table 2:** Diagnostic performance of the me*PENK* test for detection of recurrent bladder cancer

Parameter	Result
Sensitivity, % (*n*/*N*) a [95% CI]	76.3 (45/59) [63.4–86.4]
Specificity, % (*n*/*N*) b [95% CI]	85.0 (108/127) [77.6–90.7]
Positive predictive value, % [95% CI] c	70.3 [60.4–78.6]
Negative predictive value, % [95% CI] c	88.5 [82.9–92.5]
**Subgroup analyses**	
Ta, % (*n*/*N*) a [95% CI]	44.4 (4/9) [13.7–78.8]
Low grade	44.4 (4/9) [13.7–78.8]
High grade	–
Carcinoma in situ, % (*n*/*N*) a [95% CI]	91.7 (11/12) [61.6–99.8]
Low grade	–
High grade	85.7 (6/7) [42.1–99.6]
Unknown	100 (5/5) [47.8–100]
T1, % (*n*/*N*) a [95% CI]	83.3 (25/30) [65.2–94.3]
Low grade	33.3 (1/3) [0.84–90.6]
High grade	89.3 (25/28) [71.8–97.7]
T2–T4, % (*n*/*N*) a [95% CI]	62.5 (5/8) [24.5–91.5]
Low grade	–
High grade	71.4 (5/7) [29.0–96.3]
Unknown	0 (0/1)
Grade, % (*n*/*N*) a [95% CI]	
Low	41.7 (5/12) [15.2–72.4]
High	85.4 (35/41) [70.9–94.5]

CI = confidence interval.

a Number of positive *PENK* methylation results/number of test samples.

b Number of negative *PENK* methylation results/number of test samples.

c The prevalence of recurrent bladder cancer was assumed to be 31.7% based on this work.

We assessed the performance of the me*PENK* test in risk groups stratified according to the National Comprehensive Cancer Network classification [16]. In the group with low/intermediate risk, the me*PENK* test detected BC recurrence with sensitivity of 41.7% (5/12 ; 95% CI 15.7–72.3%), NPV of 95.4% (95% CI 92.8–97.1%), and PPV of 16.2% (95% CI 8.1–29.9%). For this subgroup, the sensitivity of the me*PENK* test did not significantly differ from that of the NMP22 test or cytology (*p* > 0.05). By contrast, for the high-risk group, the me*PENK* test had sensitivity of 89.7% (35/39) for detection of recurrent BC. The NPV was 96.9% (95% CI 92.5–98.8%) and the PPV was 61.5% (95% CI 51.0–71.0%). Notably, in the high-risk subgroup the sensitivity of the me*PENK* test was significantly superior to both the NMP22 test (*p* = 0.002) and cytology (*p* < 0.001), although its specificity was lower than for cytology (*p* < 0.001). Table 3 provides detailed results.

**Table 3:** Clinical performance of the me*PENK* test in comparison to NMP22 and cytology, stratified by risk group

Risk group	Test	Sensitivity, %(positive/total samples)	*p* value	Specificity, %(negative/total samples)	*p* value
Low to intermediate	me*PENK*	41.7 (5/12)	–	85.0 (108/127)	–
	NMP22	41.7 (5/12)	1.000 a	81.9 (104/127)	0.608 a
	Cytology	16.7 (2/12)	0.375 b	97.6 (124/127)	<0.001 b
High	me*PENK*	89.7 (35/39)	–	85.0 (108/127)	–
	NMP22	56.4 (22/39)	0.002 a	81.9 (104/127)	0.608 a
	Cytology	33.3 (13/39)	<0.001^b^	97.6 (124/127)	<0.001 b

a me*PENK* test versus NMP22; McNemar’s test.

b me*PENK* test versus cytology; McNemar’s test.

### 3.4 Prognostic and predictive value of the me*PENK* test

**Fig. 3:**
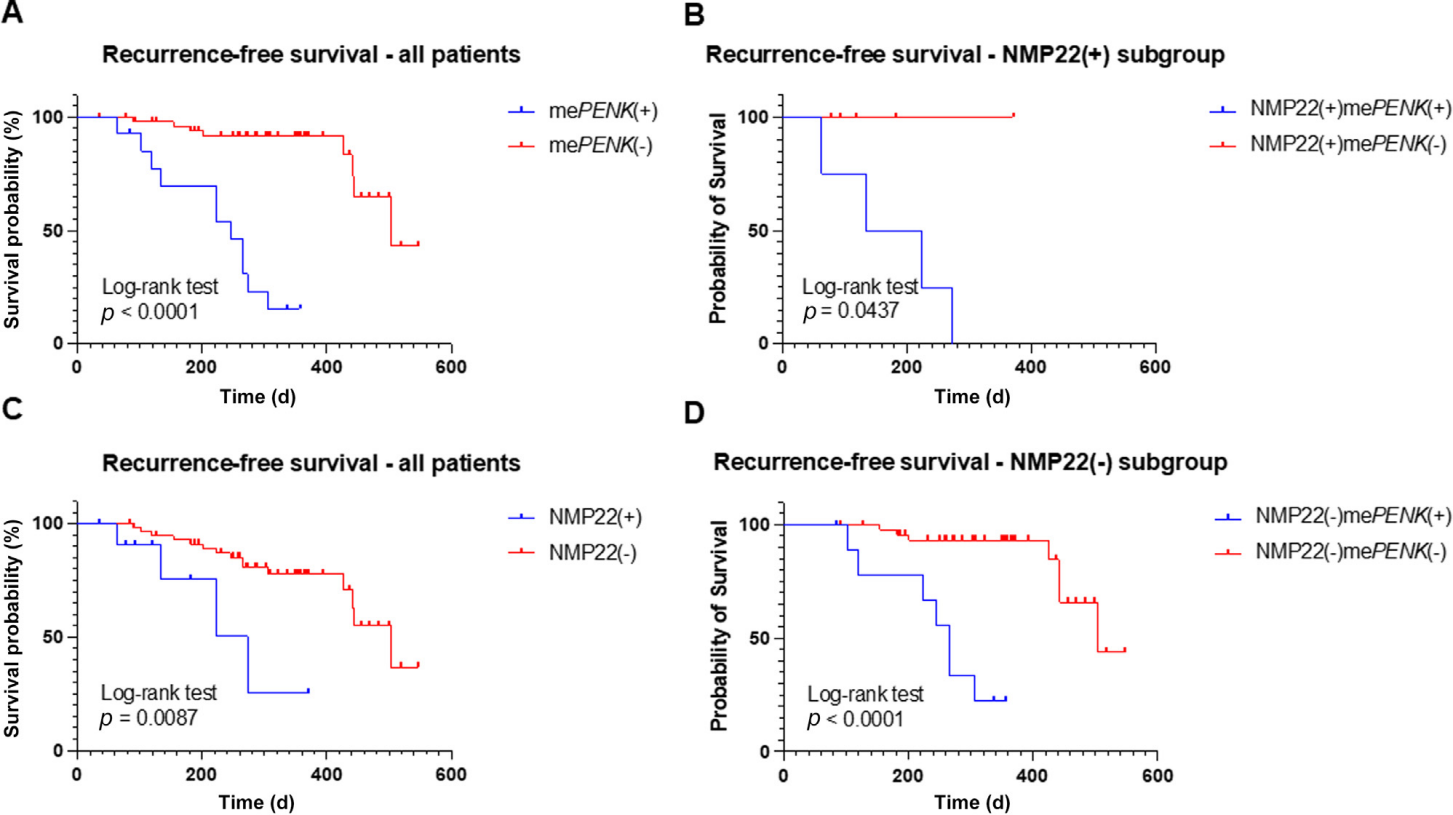
Recurrence-free survival for patients with non–muscle-invasive bladder cancer during follow-up surveillance after negative cystoscopy. (A) All patients stratified by me*PENK* status. (B) NMP22-positive subgroup stratified by me*PENK* status. (C) All patients stratified by NMP22 status. (D) NMP22-negative subgroup stratified by me*PENK* status.

We evaluated the prognostic relevance of the me*PENK* test for BC surveillance in a group of 71 patients who were recurrence-free at the time of diagnosis and had follow-up cystoscopy at median follow-up of 223 d. Of these, 19/71 patients experienced BC recurrences. Importantly, median RFS was significantly shorter for me*PENK*-positive patients (*n* = 14; 245 d) than for me*PENK*-negative patients (*n* = 57; 503 d), with a HR of 9.4 (95% CI 2.4–35.9; *p* < 0.001, log-rank test; Fig. 3A). In detail, of the 14 me*PENK*-positive patients, 11 (78.5%) had BC recurrence, while only eight of 57 me*PENK*-negative patients (14.0%) experienced BC recurrence. This result indicates that patients with positive me*PENK* results had a significantly higher risk of tumor recurrence. In addition, if we assume that these patients had BC recurrence at the time of me*PENK* testing, the overall sensitivity for detection of BC recurrence is 80.0% (56/70), with specificity of 93.1%.

The urinary NMP22 test also had prognostic power for RFS in BC (Fig. 3B). However, regardless of the NMP22 result, RFS was significantly shorter for me*PENK*-positive than for me*PENK*-negative patients. This result indicates that the me*PENK* test had better prognostic power than the NMP22 test (*p* = 0.0437 for the NMP22^+^ subgroup; *p* < 0.0001 for the NMP22^−^ subgroup, log-rank test; Fig. 3C,D).

## 4 Discussion

We previously demonstrated that the me*PENK*-LTE/qMSP test has promising potential for initial diagnosis of primary BC among patients with hematuria [15]. The findings from the present study may add value to the potential clinical applications of the modified me*PENK* test beyond initial diagnosis, specifically in the context of monitoring for BC recurrence.

Cystoscopy with urine cytology is the standard method used for BC surveillance. Although cystoscopy is effective, it is expensive and invasive. Moreover, its sensitivity varies between 62% and 84%, depending on the tumor type, stage, and grade [17]. Urine cytology remains a prevalent approach for BC diagnosis and surveillance, but it has shortcomings in terms of sensitivity [6]. The utility of the urinary NMP22 test for NMIBC diagnosis and surveillance has been demonstrated, with sensitivity and specificity values for detection of primary or recurrent BC ranging from 49.5% to 55.7%, and from 85.7% to 87.3%, respectively [18], [19], [20].

Previous studies directly compared the ability of urine biomarkers, cytology, FISH, uCyt+, and NMP22 tests to predict the risk of NMIBC recurrence and progression during surveillance. Patients with biomarker-positive findings but negative cystoscopy have a higher risk of recurrence and progression; for patients with negative cytology, only NMP22 remains predictive for recurrence [21]. Therefore, we chose cytology and the NMP22 test as a reasonable benchmark to evaluate the me*PENK* test for detection of BC recurrence during surveillance. The overall sensitivity of the me*PENK* test for detection of BC recurrence was 76.3%, with specificity of 85.0% and an NPV of 88.5%. The test had higher sensitivity for the high-risk group, at 89.7%, with an NPV of 96.9%. Notably, the me*PENK* test detected CIS with high sensitivity of 91.7%.

In general, when considering the intended use of a test for BC monitoring to exclude recurrence during follow-up, higher NPV is often considered more practical. Our study included 29 healthy individuals in the training set to establish the cutoff value, and only six of these exhibited a signal, ranging from 31.0 to 26.7. It is noteworthy that adjusting the cutoff from the original 32.0 to a lower value may result in more favorable test outcomes. For example, setting the cutoff to 26.7 yields sensitivity for the high-risk group of 100%, with a corresponding NPV of 100%. While this adjustment holds potential for allowing individuals with a negative result to forego invasive cystoscopy examinations, it is important to acknowledge that this improvement comes at the expense of specificity, which decreased to 38.6%. Therefore, a prospective clinical trial with a large-scale longitudinal design is imperative to ascertain the optimal cutoff that balances effectiveness and utility for use in clinical practice. Further research will contribute to refining the test performance and ensuring its reliable application in recurrence monitoring for BC.

The sensitivity of the me*PENK* test for detection of BC recurrence in the subgroup with low/intermediate risk was 41.7%. The relatively low sensitivity for this subgroup may be attributed to the small size or to the adherent nature of the recurrent tumors; at this stage, the tumors might not actively shed a detectable number of tumor cells into the urine [22]. Patients with BC in the high-risk group have a lifelong risk of disease progression and mortality. Patients in the low-risk group have very low risk of progression: the 15-yr progression-free survival rate is 95%, with no cancer-specific mortality [23], [24]. Thus, in the clinical setting, identification of patients with high-risk BC is more critical for reduction of disease-related morbidity and mortality. In the high-risk setting, the specificity of the me*PENK* test was similar to that of the NMP22 test, but inferior to the specificity of cytology.

Molecular tests such as Xpert Bladder (five mRNA markers), CxBladder (five mRNA markers), AssureMDx (three gene mutations and three gene methylations), and Bladder EpiChek (15 gene methylations) are available for BC surveillance. These tests have sensitivity ranging from 68% to 82%, and specificity ranging from 80% to 88% [25]. Our findings are comparable to results from clinical studies using these tests. Nonetheless, high-level evidence obtained from large-scale clinical studies is needed before biomarker-based urine tests can be adopted in routine clinical practice.

Only a few studies have evaluated the prognostic value of urine markers in follow-up surveillance for NMIBC, and the effectiveness of these markers has not been proven [21]. Here we analyzed the prognostic relevance of the me*PENK* test for BC recurrence. Follow-up cystoscopy surveillance for cystoscopy-negative patients revealed that median RFS was significantly shorter for me*PENK*-positive than for me*PENK*-negative patients. This finding indicates that me*PENK*-positive patients had a significantly higher risk of tumor recurrence. The group with positive me*PENK* results had significantly shorter median RFS, regardless of NMP22 results. This finding indicates that the urinary me*PENK* methylation test performed better for prediction of BC recurrence than the NMP22 test.

Among 71 patients with negative cystoscopy results, 19 experienced a subsequent recurrence. Importantly, 11/19 patients (57.9%) had a positive me*PENK* test at the time of baseline cystoscopy, which confirms that the me*PENK* result was predictive of recurrence, as validated via follow-up cystoscopy [26]. If we consider these 11 patients as having recurrent BC, the overall sensitivity and specificity for detection of BC recurrence improved to 80.0% and 93.1%, respectively. These findings suggest that a positive me*PENK* result is a useful tool for identification of patients with a high likelihood of BC recurrence.

Our study has some limitations, such as the small sample size, single-site enrollment, and short follow-up. Healthy individuals were used as the control group when determining the cutoff value for the test, and there is an age disparity between this group and the BC patients. Nevertheless, the performance findings provide valuable information and warrant validation of the me*PENK* test in a large-scale prospective study.

## 5 Conclusions

In our study, the urinary me*PENK* test demonstrated notable sensitivity in detecting recurrent BC, especially for high-risk cases, surpassing the performance of NMP22 and urine cytology. A positive me*PENK* result correlated significantly with shorter RFS times and accurately predicted recurrence following cystoscopy. This finding underscores the potential of the me*PENK* test as a valuable tool for providing prognostic and predictive information in BC surveillance. Our study suggests that patients with negative me*PENK* results may benefit from extended intervals between cystoscopies in the surveillance of NMIBC. Use of the urine-based me*PENK* test with its NPV holds promise for reducing unnecessary cystoscopy procedures during follow-up for patients with negative results. Furthermore, lowering the cutoff value could increase the test sensitivity and maximize its NPV, with potential to exclude BC more effectively. Acknowledging that the optimal cutoff value may vary depending on the clinical context and objectives is crucial. Large-scale longitudinal clinical studies are imperative to validate and integrate these proposed adjustments into routine clinical practice.

  ***Author contributions:*** Young-Deuk Choi had full access to all the data in the study and takes responsibility for the integrity of the data and the accuracy of the data analysis.

  *Study concept and design*: Han, An, Choi.

*Acquisition of data*: Heo, J. Lee, Jang, S.H. Lee, Ham, Hwang, Choi

*Analysis and interpretation of data*: Han, Oh, Hwang

*Drafting of the manuscript*: Han, Oh.

*Critical revision of the manuscript for important intellectual content*: Han, Oh, An.

*Statistical analysis*: Han, Oh.

*Obtaining funding*: Han.

*Administrative, technical, or material support*: Oh, JH, An

*Supervision*: Choi.

*Other*: None.

  ***Financial disclosures:*** Young-Deuk Choi certifies that all conflicts of interest, including specific financial interests and relationships and affiliations relevant to the subject matter or materials discussed in the manuscript (eg, employment/affiliation, grants or funding, consultancies, honoraria, stock ownership or options, expert testimony, royalties, or patents filed, received, or pending), are the following: Jaehee Hwang is an employee of Genomictree, Inc. Tae Jeong Oh and Sungwhan An are employees of and hold shares in Genomictree, Inc. The remaining authors have nothing to disclose.

  ***Funding/Support and role of the sponsor:*** This study was sponsored by Genomictree, Inc. and by a Korea Health Industry Development Institute grant for research (HI22C118600; Principal Investigator Hyunho Han). The funding body had a role in the study design, data collection, analysis, and interpretation, and manuscript preparation.
