# Supplementary material for: Clinical Validation of the Proenkephalin (PENK) Methylation Urine Test for Monitoring Recurrence of Non–muscle-invasive Bladder Cancer
Source: Eur Urol Open Sci. 2024 Mar 7;62:99–106. doi: 10.1016/j.euros.2024.02.010 (PMC10940910; doi:10.1016/j.euros.2024.02.010)
Supplement: Supplementary data 1 [file mmc1.pdf]

Supplementary Figure 1

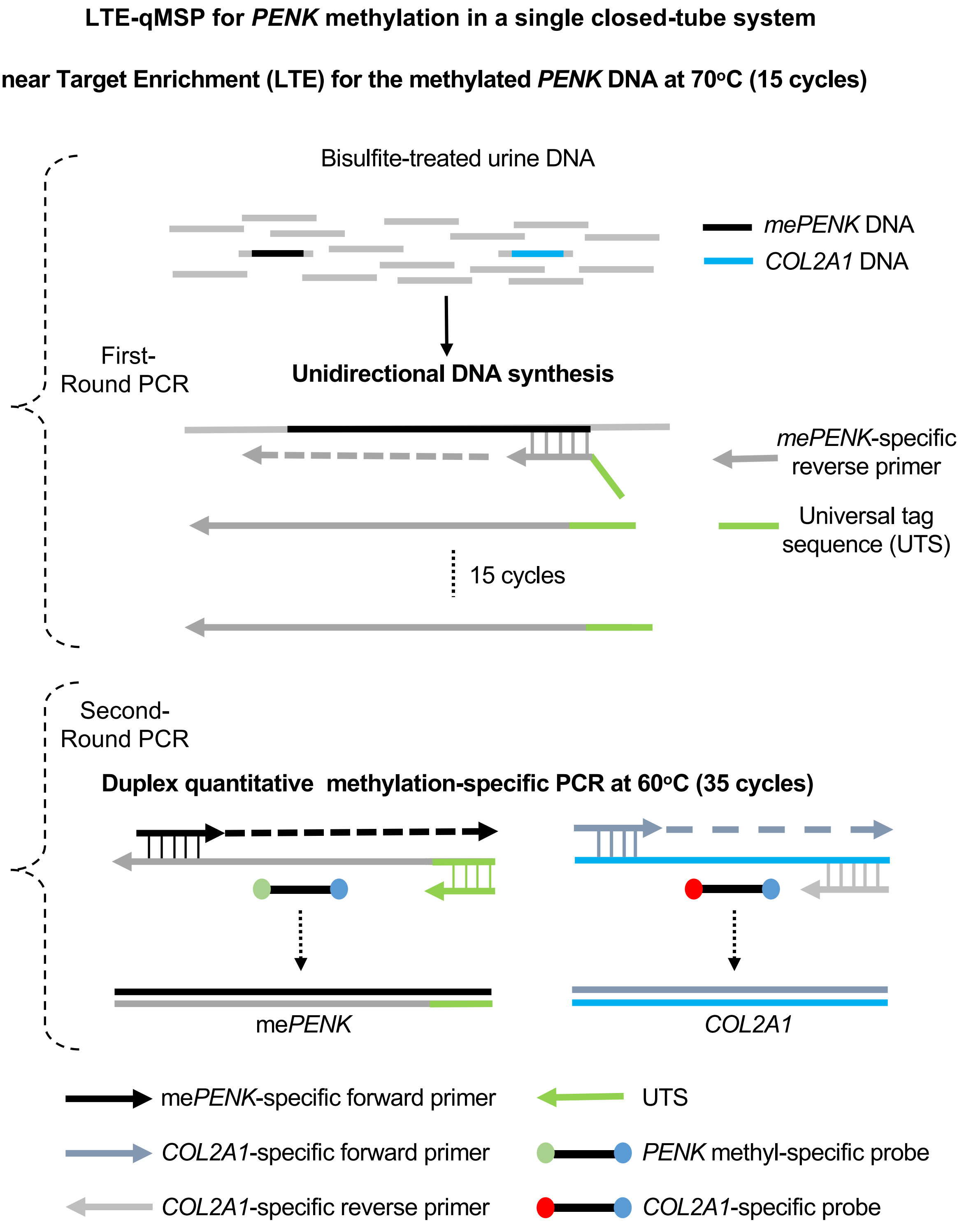

Supplementary Figure 1. Outline of a one-step mePENK-LTE-qMSP assay. The assay comprises two rounds of amplification step, sequentially occurring in a single tube. The first round is the LTE (at 70oC) using anti-sense primer attached to a universal tag sequence to enrich methylated PENK DNA. The second round is duplex qMSP (at 60oC) and generates fluorescent signals at two distinct wavelengths.
