# Supplementary material for: Clinical Validation of the Proenkephalin (PENK) Methylation Urine Test for Monitoring Recurrence of Non–muscle-invasive Bladder Cancer
Source: Eur Urol Open Sci. 2024 Mar 7;62:99–106. doi: 10.1016/j.euros.2024.02.010 (PMC10940910; doi:10.1016/j.euros.2024.02.010)
Supplement: Supplementary data 2 [file mmc2.docx]

**Supplementary Table 1.** Sequences of primers and probes used in me*PENK* test

| **Target gene** | **Primers and probes** | **Sequence, 5’ to 3’^a^** |
| --- | --- | --- |
| *PENK* | Sense | GGTGTTTTAGGTAGTTTCGC |
|  | Anti-sense | AAATAAACGTAAAAACGACTCAAATCGCCTCGC G |
|  | Probe | FAM-TGGGGGCGATCGCGT TATTTCGG |
| *COL2A1* | Sense | TAGGAGTATTAGTAATGTTAGGAGTA |
|  | Anti-sense | CTACCCCAAAAAAACCCAATCC |
|  | Probe | Cy5-AGAAGAAGGGAGGGGTGTTAGGAGAGG |
| Unique tag sequence |  | ACTGATAAGGCGACCACCGA |

^a^Underlines indicated CpG dinucleotide sites.
